# Integrating design thinking with sustainability science: a Research through Design approach

**DOI:** 10.1007/s11625-018-0618-6

**Published:** 2018-08-20

**Authors:** Ray Maher, Melanie Maher, Samuel Mann, Clive A. McAlpine

**Affiliations:** 1 School of Earth and Environmental Science, The University of Queensland, Brisbane, QLD Australia; 2 Centre for Policy Futures, The University of Queensland, Brisbane, QLD Australia; 3 Visual Communication Design, Brisbane, Australia; 4 Otago Polytechnic, Dunedin, New Zealand

**Keywords:** Research through design, Sustainability, Design thinking, Interdisciplinary collaboration, Conceptual frameworks, Visual communication, Social–ecological systems, Cross-scale interactions

## Abstract

Design disciplines have a long history of creating well-integrated solutions to challenges which are complex, uncertain and contested by multiple stakeholders. Society faces similar challenges in implementing the Sustainable Development Goals, so design methods hold much potential. While principles of good design are well established, there has been limited integration of design thinking with sustainability science. To advance this integration, we examine the process of designing MetaMAP: an interactive graphic tool for collaborating to understand social–ecological systems and design well-integrated solutions. MetaMAP was created using Research through Design methods which integrate creative and scientific thinking. By applying design thinking, researchers and practitioners from different backgrounds undertook multiple cycles of problem framing, solution development, testing and reflection. The testing was highly collaborative involving over 150 people from diverse disciplines in workshops, case studies, interviews and critique. Reflecting on this process, we discuss design principles and opportunities for integrating design thinking with sustainability science to help achieve Sustainable Development Goals.

**Electronic supplementary material:**

The online version of this article (10.1007/s11625-018-0618-6) contains supplementary material, which is available to authorized users.

## Introduction

This paper describes the application of design methodology to sustainability goals. The United Nations’ Sustainable Development Goals (SDGs) provide a common direction (Griggs et al. 2013) for guiding collaborating towards a sustainable future. However, several conceptual, institutional, and communication barriers restrict our progress in achieving SDGs in a cohesive fashion (Maher et al. 2018b). Integrating design-based approaches with sustainability science may help to overcome many of the current challenges limiting progress towards SDGs. We propose that Research through Design (Zimmerman et al. 2010) is well suited to achieve sustainability goals by applying design approaches in a research context. Research through Design solves complex and contested challenges by taking a holistic approach and developing ideas through many iterations of proposition and critical reflection (Glanville 2007). To give this design approach some context, we describe the process of designing MetaMAP as a case study.

### Case study introduced

**Fig. 1:**
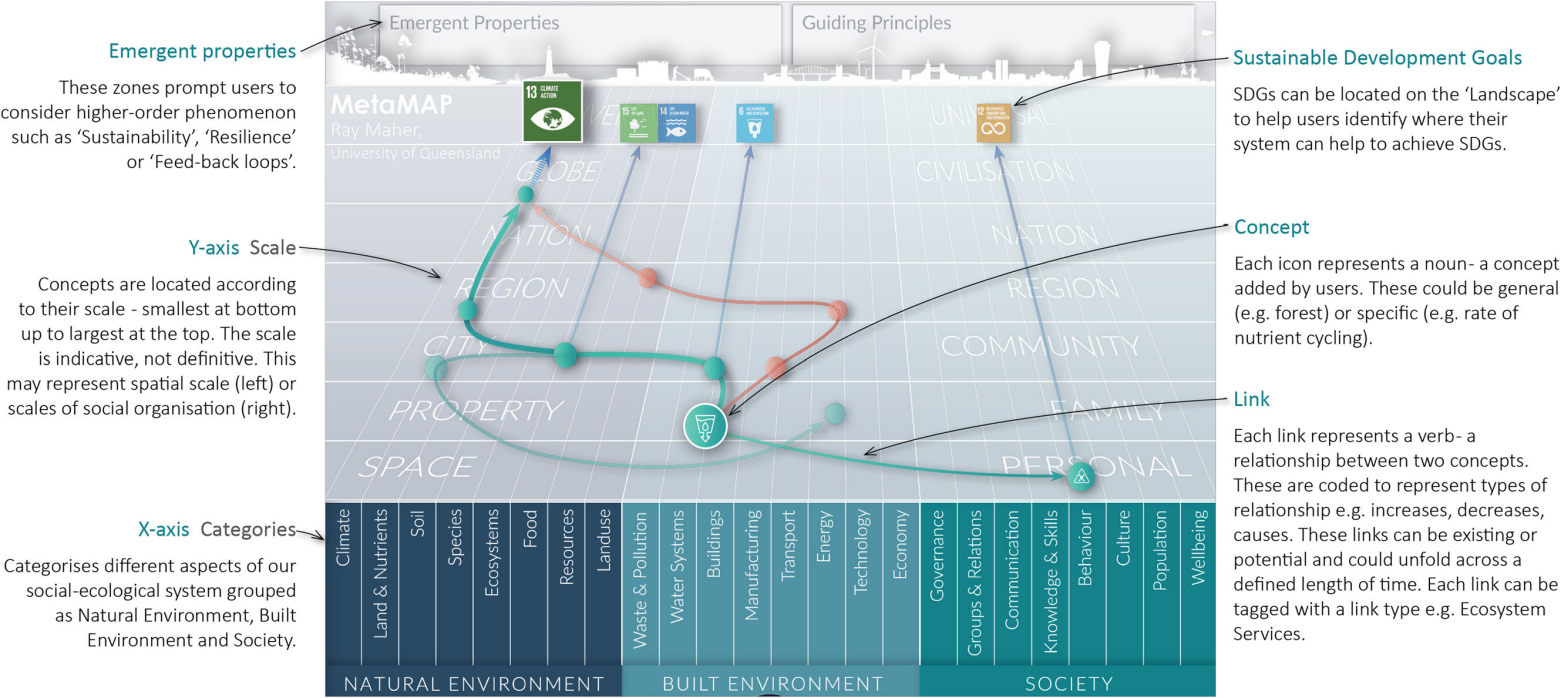
MetaMAP framework notated to identify core elements. The intent of this paper is not to describe MetaMAP in detail, rather to use its derivation as a case study in the application of the design thinking approach in addressing Sustainable Development Goals

MetaMAP is an interactive graphical tool which supports collaborative investigation and design for achieving SDGs. It helps diverse users to integrate their thinking, understand sustainability challenges holistically, and develop well-integrated solutions (Fig. 1). A detailed description with worked examples and further applications can be found in Maher et al. (2018a). In brief, the structure and application of the MetaMAP framework help users gain insight by seeing relationships among parts of the natural environment, built environment and society across multiple spatial and temporal scales. It provides an inclusive framework to help people from different backgrounds integrate their diverse perspectives on sustainability issues into a common understanding. Armed with this holistic perspective, guided process help users to identify points of leverage and design well-integrated sustainability initiatives.

### Background

Global society faces substantial challenges in transforming our relationship with the natural environment. To support this transformation, the United Nation’s Sustainable Development Goals (SDGs) provide a common direction (Griggs et al. 2013). However, our traditional approach for building knowledge and solving problems is poorly suited to the unique nature of sustainability challenges (Maher et al. 2018a; Sterling 2009). Sustainability problems are complex and contentious and transcend the boundaries of disciplines and nations (Brandt et al. 2013). Many current institutional structures (e.g. strict hierarchies) and thinking paradigms (e.g. reductionist thinking and reliance on single metrics) lead us to look at problems in isolation (Siebert 2011). This causes many sustainability initiatives conceived in theory to fail in practice due to issues outside the scope of consideration. Sustainability initiatives designed in isolation lack synergy, so advances in one area may setback others. This siloed approach makes it difficult to build wide support for initiatives which only address narrow interests.

Growing concern with the isolated approaches often used for achieving SDGs is prompting calls for more integrated approaches. The necessary integration takes several forms, including understanding the context of sustainability challenges more holistically, connecting people and ideas across social and institutional divides (Khalili et al. 2017), and understanding interactions among SDGs (Stafford-Smith et al. 2017). The aim of such an approach is to produce sustainability initiatives which are more effective, more efficient and better aligned with diverse interests.

To support this, researchers have developed more integrated conceptual frameworks for holistically understanding sustainability challenges and their context. By organising concepts meaningfully, these frameworks help to guide enquiry and provide a foundation for stronger collaboration across disciplines (Heemskerk et al. 2003). Several well-established frameworks have become a foundation of research and education in sustainability science including resilience (Berkes and Ross 2013; Folke 2006), planetary boundaries (Rockström et al. 2009) and ecosystem services (Abson et al. 2014; Bennett et al. 2009). Partelow (2015) calls for a more thorough and deliberate integration of Ostrom’s social–ecological systems framework (Ostrom 2009) with sustainability science. In contrast to other highly technical and rigid frameworks, Hall et al. (2017) translate Luhmann’s social system theory to the development of sustainability solutions. Of particular interest is the concept of resonance—the “sweet spot…[where] all the factors…come together beautifully” (Hall et al. 2017). It represents a convergence of mutually reinforcing feedback loops across multiple sectors of society. This can be applied strategically to align the interests of diverse stakeholders which is critical for long-term success of sustainability initiatives. We examine several of these frameworks, their benefits and limitations elsewhere (Maher et al. 2018a).

Building on these frameworks, many tools have been developed to support decision making for sustainability. Recent developments in this area include the SDG Interlinkages Framework (Nilsson et al. 2016) which helps to understand typical interactions among selected SDGs at a general level. This supports the argument for achieving SDGs in an integrated fashion. However, it may be of less value for informing specific sustainability initiatives where actual conflicts and Synergy depend on unique social, ecological and political circumstances. The System Dynamics-based iSDG family of models is tools for comparing and choosing among competing sustainability initiatives (Collste et al. 2017). This is valuable for maximising multiple known sustainability outcomes in alignment with stakeholder demands. However, the initiatives being assessed must be predetermined using other means. The graphical multi-agent decision-making model (GMADM) (Khalili et al. 2017) also depends on assumed plans or portfolios. While the tool is well formulated, focusing on comparative analysis has limited value for challenging assumptions or generating new ideas on which to build creative and innovative solutions. Tools that prioritise analysis restrict lateral thinking—our ability to perceive and re-conceptualise things in fundamentally new ways (de Bono 1970). This means outcomes tend to optimise and reinforce existing ways of being rather than transformation. In short, these are tools for analysis, not design. The demands on tools for addressing SDGs are tremendous. They must: aggregate knowledge across disciplines (Wiek et al. 2012; Partelow 2015), educate users in systems thinking (Stafford-Smith et al. 2017), bridge across geographical and political scales (Collste Collste et al. 2017), link theory with policy (Khalili et al. 2017), incorporate social–ecological systems with ecosystems services (Partelow 2015), link theory with real-world projects (Lang et al. 2017), connect intellectual concepts with shared social values (Lang et al. 2017; Hall et al. 2017) and promote new ways of thinking (Hall et al. 2017). To be widely used, tools for addressing SDGs must be intuitive, practical and suitable for a wide diversity of users with limited specialist training. Underlying these requirements is a need for tools which support the “creative coordination of resources, capacities, and information into new ways of seeing the system which are useful for designing strategic interventions in the setting” (Hall et al. 2017). While new tools are of value for strategic decision making, they do little to support the creativity and design required.

### The need to advance design approaches for achieving sustainability goals

Integrating design-based approaches with sustainability science may help to overcome many of the current challenges limiting progress towards SDGs. While these types of ‘wicked’ challenges (Bojórquez-Tapia et al. 2017) are relatively new to science, other disciplines face similar types of challenges and have well-established methods for doing so. Design disciplines, especially architecture, commonly face “…incalculably complex (and ambiguously defined) problems, bringing them to simple resolution: designers typically make one object that satisfies a myriad of often contradictory and ill-defined requirements” (Glanville 2007). Architects have highly developed techniques for making sense of complex situations, generating innovative strategies for solving problems, integrating multiple perspectives and achieving many goals simultaneously (Dorst 2011; Rodgers and Yee 2014). These both complement and are supported by recent advances in sustainability science (Nassauer and Opdam 2008). Integrating design approaches with sustainability science offers substantial opportunities for achieving SDGs efficiently and effectively amid real-world complexity.

Despite calls for more design approaches to sustainability research and practice (Future Earth 2014; Kolko 2009), design approaches are rare among research on solutions for the SDGs. This is likely because design is seen as mysterious, even ‘magical’ (Glanville 2007) by those who are unfamiliar with its methods. It also follows a different logic and methodology to the sciences which have dominated our progress in sustainability. However, design and sustainability science are highly compatible and integrating them can produce sustainability initiatives which are effective, transformational, and well integrated into their unique social–ecological context. The contribution of this paper is to go beyond that magic by introducing the attitudes, processes and principles that make design work.

To advance this integration, sustainability researchers and practitioners need a better understanding of design principles, design methods, and their value for supporting sustainability science. In this paper, we provide an overview of Research through Design methodology, its value for achieving SDGs and its compatibility with sustainability science. We then examine a case study of a Research through Design project which develops new tools for achieving SDGs. We then unpack the design process for easier comprehension and integration with sustainability science. We describe five stages of design, each a cycle of: (a) problem framing, (b) solution development, (c) testing and (d) critical reflection. We then briefly describe the primary research outcome: MetaMAP—a graphical tool for collaborating to understand social–ecological systems holistically and design well-integrated initiatives (described in detail in Maher et al. 2018a). Reflecting, we distil some core design principles applied in the case study and discuss implications for their wider application to achieve SDGs. Finally, a brief conclusion identifies limits and opportunities to extend both Research through Design methodology and MetaMAP.

## Research through design methodology

Research through Design (RtD) translates methods and mental processes from design practice to a research environment (Zimmerman et al. 2010). Design is a process of producing simple and effective responses to complex and vague problems that span across disciplines and stakeholder groups. Design has been described as a process of “…reflection-in-action” (Kennedy-Clark 2013), and as “…organizing complexity or finding clarity in chaos…” (Kolko 2009). It takes a holistic perspective, drawing together different perspectives on problems and their context, technology, human needs, empathy with users and stakeholders to create aesthetic artefacts, which can be rich in meaning.

Research through Design offers many advantages for research on sustainability and achieving SDGs. By taking a holistic perspective and expanding the framing of the context of the problem, RtD methods can create better integrated sustainability initiatives—where components work in harmony with each other and their context. Developing a proposal through multiple iterations can help a single initiative to achieve multiple goals simultaneously. By focusing on synthesis over analysis, RtD can create usable artifacts, fit for a specific time and place in the real world. Engaging diverse stakeholders in a project enriches outcomes and helps to secure wider support for its findings. Each of these approaches helps to address critical limits to progress on the SDGs. These principles are demonstrated in practice through the case study of MetaMAP. At the end of this article, we discuss some fundamental design principles of particular relevance to achieving SDGs. More detail can be found elsewhere (Faste and Faste 2012; Kennedy-Clark 2013; Moloney 2015; Rodgers and Yee 2014; Zimmerman et al. 2007, 2010), but usually without specific reference to sustainability.

Table 1 provides a summary of some typical differences between the focus of traditional scientific and design approaches to research (Hes and Du Plessis 2014; Sterling 2009). Readers will see that sustainability science is shifting towards the right column. We will demonstrate that this evolution can be accelerated by integrating design and sustainability science.

**Table 1:** Differences between the typical focus of traditional mechanistic and design approaches to research

Traditional mechanistic approach	Design thinking
Describe	Transform
Analyse	Synthesise
Generally applicable	Context specific
Narrow problem framing	Broad opportunity seeking
Past focused	Future focused
Optimising existing systems	Inventing new systems
Grounded in deductive reasoning	Grounded in abductive reasoning (Kolko 2009)
Progress through concrete incremental steps	Progress through insightful jumps followed by critical reflection

### Design principles

There are several design principles which help designers to solve wicked problems and can help to achieve SDGs. These principles act as rules of thumb and attitudes which help to guide design processes towards innovative, valuable, and well-integrated outcomes (e.g. Rodgers and Yee 2014). Five design principles of particular value to achieving SDGs are: broad problem framing supporting multiple goals; maximise synergy, minimise compromise; integrating diverse perspectives; thinking visually; and multiple feedback loops. These are demonstrated throughout the case study below and expanded in detail following it.

## Case study: designing MetaMAP using a research through design approach

We now examine a case study, which applies Research through Design methods to develop MetaMAP: a graphical tool for achieving SDGs. This begins with an overview and structure of the Research through Design process. We then follow the narrative of designing MetaMAP through five stages, each containing four types of design activity. Throughout this process, we recorded how the design developed, feedback from collaborative testing and our own critical reflections on the process. We end this section with an overview of MetaMAP, who it is for and how it works. In total, this provides a successful demonstration of design methods for research supporting SDGs and a vehicle to discuss methodological development and broader applications.

### MetaMAP aims

It is not possible to strictly predefine the aims of an RtD project as aims continuously evolve in response to new understandings gained as the design advances. Initially, we aimed to design a digital platform for integrating and sharing an ecosystem of knowledge and action for sustainability. As the design process uncovered new opportunities and needs, our aims shifted to creating graphical tools for collaborating to understand and visualise social–ecological systems and design well-integrated initiatives to achieve SDGs. This reframing is evident in the narrative below.

### Overview and structure of the research through design process

**Fig. 2:**
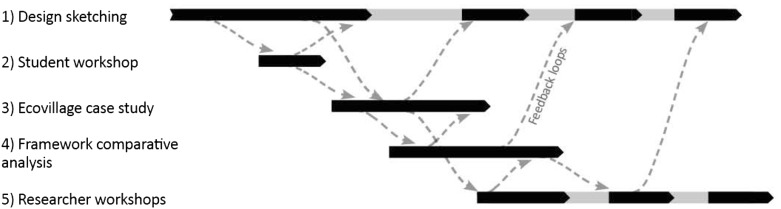
Five parallel stages of design with multiple avenues of feedback

Designing MetaMAP was a significant undertaking which addressed uncertain, vaguely defined and conflicting goals and a scope which transcended academic disciplines. To synthesise this complexity into harmonious resolution, we developed MetaMap using Research through Design methodology. The design was advanced through several stages, each building on the previous. The stages were advanced simultaneously so that progress in one aspect could inform others (Fig. 2). This approach is a common design strategy and was considered superior to sequential stages which remove the possibility of feedback loops (Moloney 2015). We have described the process as a linear flow due to the limits of text. However, the process is non-linear and the reader should be aware of the integration and interaction of these stages.

**Fig. 3:**
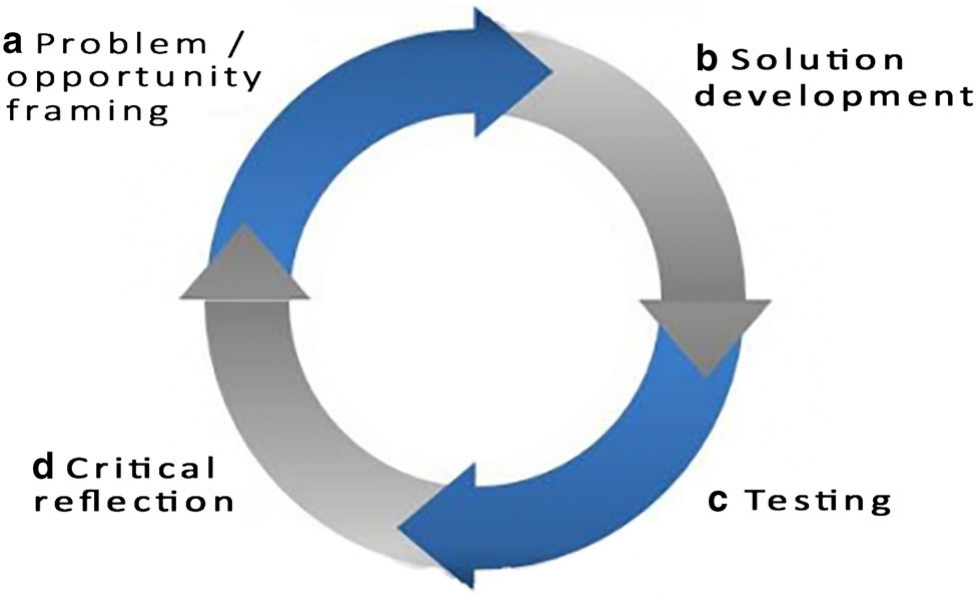
Each stage can be considered a cycle of four design activities

Each stage can be considered as a cycle of four design activities: (a) problem/opportunity framing, (b) solution development, (c) testing and (d) critical reflection as shown in Fig. 3. Each cycle helps to “…re-define the problems, possible solutions, and the principles that might best address them” (Amiel and Reeves 2008). In practice, these design activities were not strictly predefined in order to take advantage of inspiration and opportunities for critique as they arose. This agile process involving many layers of feedback loops is a fundamental principle of design for addressing wicked problems (Faste and Faste 2012). It has significant value for achieving SDGs amid real-world complexity. We now introduce the four types of design activities in each cycle.

#### Activity type a: problem/opportunity framing

The first design activity in each cycle involved framing the problem, identifying opportunities and (re)defining limits. The designers considered questions such as:

- What issues should we be considering?
- How might they relate?
- How have similar challenges been addressed elsewhere?
- Where are the synergies trade-offs and priorities in this unique case?
- Who are the stakeholders (in the broadest sense)?
- What other benefits might we gain beyond our initial objectives?
- What other disciplines might provide guidance?

The specific methods used in this design activity varied in each stage. Common activities included: reviewing literature, semi-structured interviews, analysing precedents, concept mapping, pin boards and writing design briefs.

#### Activity type b: solution development

**Fig. 4:**
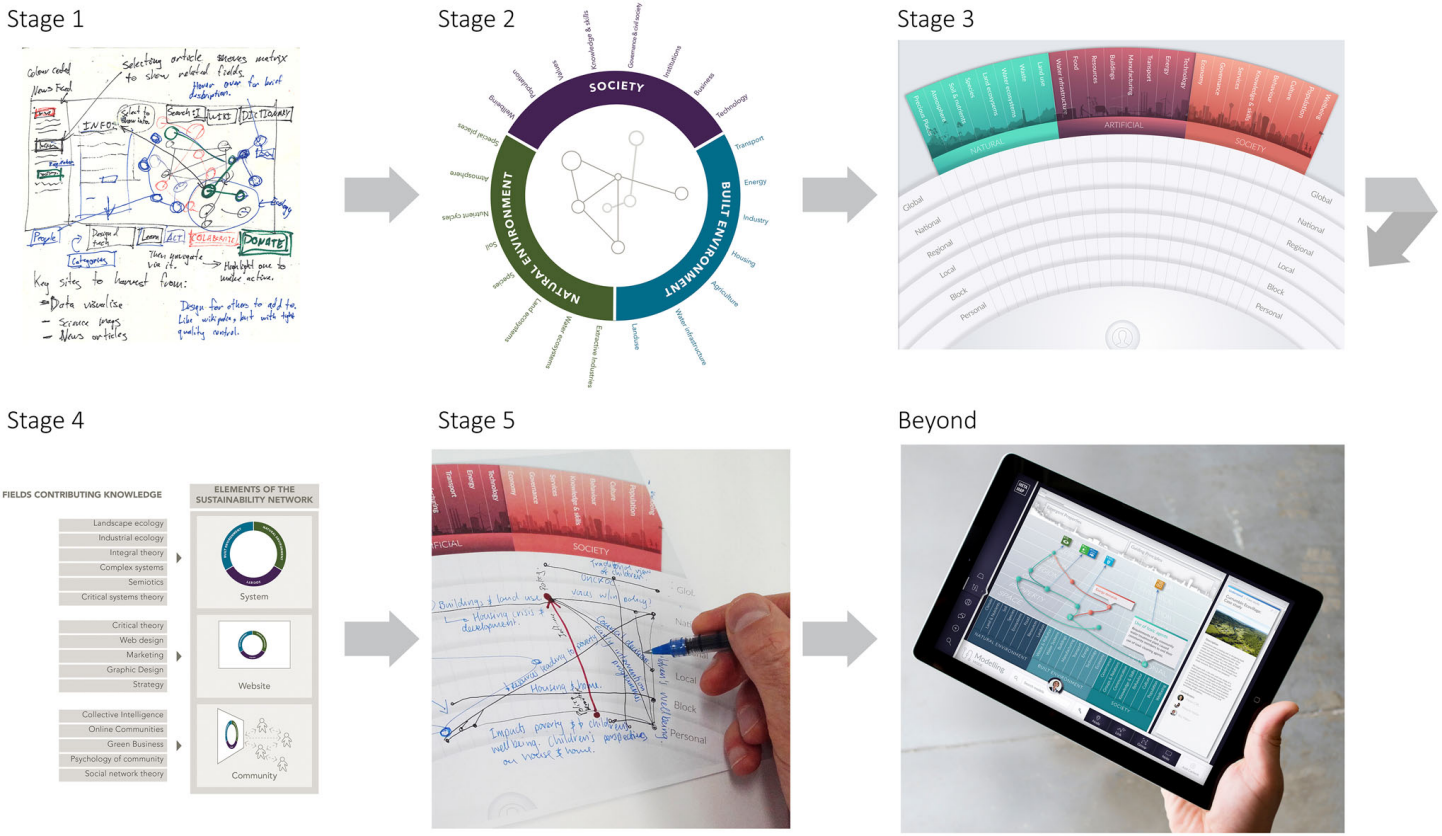
Overview of design concepts in the evolution of MetaMAP from sketch to designed digital interface (details of each stage given in text and figures in following sections)

Based on framing of the problem/opportunity space, we designed possible solutions. We represented concepts visually through sketches, diagrams, paper-based prototypes and digital mockups. Methods for generating and developing innovative ideas varied greatly throughout the project. Brainstorming was also used to produce many concepts rapidly without prejudging them. When design ideas became stagnant or lacked originality, we applied lateral thinking approaches to reinvigorate the process (de Bono 1970). This involved browsing diverse collections of semi-related images and adapting ideas from existing precedents to new applications. Concept mapping helped us to see existing and potential relationships between different objectives and strategies. The images we created helped to visualise possible implications of our ideas and to communicate ideas to others for testing, application and critique. Figure 4 provides an overview of how MetaMAP evolved across five stages of design.

#### Activity type c: testing

One purpose for testing design prototypes was to understand which aspects of the tool were effective, which were not, and to identify fruitful opportunities for further development. Even more important was to discover the unknown unknowns—the problems and opportunities we did not know to look for. For this reason, it was important not to restrict the type of feedback we could receive from the testing process.

Most of the testing activities were collaborative involving workshops, case studies and semi-structured interviews. In total, these involved over 150 people from diverse disciplines including: Ecology, Human geography, Earth Sciences, Architecture, Environmental management, Educational psychology, Participatory GIS, Business, Human–Computer Interaction (HCI), Sustainable practice and Conservation Biology. In these tests, users applied the MetaMAP prototype in different ways to sustainability projects which varied greatly in scale and type. We observed the activities and took notes during and immediately afterwards. We also invited workshop participants to comment directly on the MetaMAP framework and its application.

#### Activity type d: critical reflection

Collaborative testing helped to validate our assumptions about how potential users would use MetaMAP. Following the tests we asked: How did participants respond to the framework? Where were they uncertain about its use? What did they find most valuable? What insights into their own work did it help them to gain? Sometimes it became clear that a previously rejected option would have performed better.

By reflecting critically on the tests, we learned about the problems and opportunities which helped to refine our judgement in making design decisions. Skilful and informed judgement is an essential part of the process. As such, design is a subjective, not objective undertaking—it involves pursuing a deliberate intention to shape the future based on a set of values. The designer is part of the process and not distinct from it.

## Case study narrative: five stages of design

We now describe each stage of design in turn. For each we outline: (a) how the problem and opportunities were framed at that time; (b) how the design evolved in response; (c) how we tested it, including main objectives and who was involved in the task undertaken; and (d) critical reflections on the design and testing.

### Stage (1) Design sketching

#### (1a) Problem/opportunity framing

Creating the right circumstances to foster inspiration is an important part of creative endeavours, including science (Scheffer 2014). MetaMAP began when I (First Author), as a recent graduate confronted by the potential and challenges of the SDGs, went for a long walk alone thinking about the future. I wanted to find the best way I could to help build a sustainable future. I knew of many sustainability challenges and opportunities to contribute, but I did not know how they were related or where I could have the biggest impact. I thought that many others must be having similar challenges at different scales, whether planning a career, designing multinational policy or just choosing which product to buy. In response, I imagined a digital world which showed how all parts of our social–ecological system were related like a giant network—constellations of ideas which I could explore to find where I could have greatest influence over a sustainable future. If we could build this digital world, what would it contain, how could it be navigated, and who would use it?

#### (1b) Solution development

**Fig. 5:**
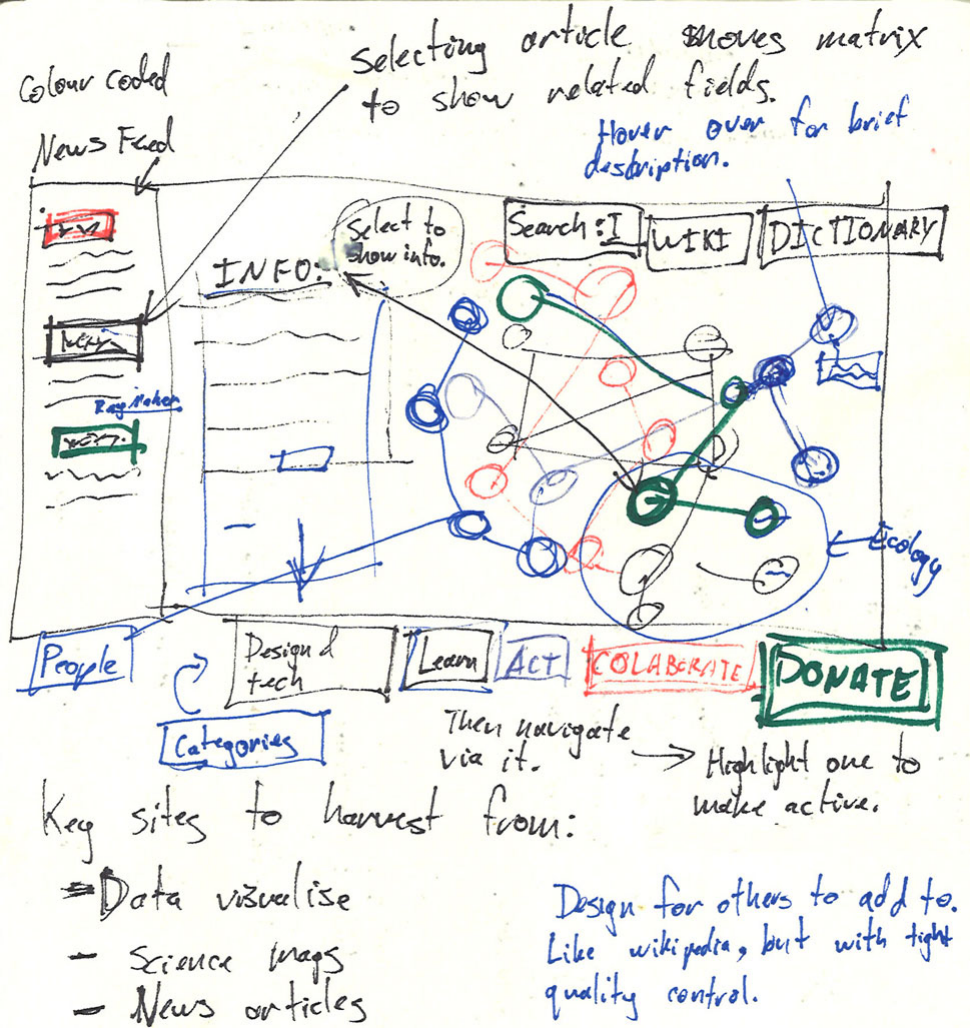
The first concept sketch

In its first conception, the design took the form of a colour-coded system model which showed relationships among diverse content on sustainability (Fig. 5). Content was to be added by users and organised by how it is acted upon: learn, act, collaborate and donate. The systems model was considered as an interactive interface which would reorganise itself as users explore chains of interaction among issues. Selecting a topic would access detailed information contributed by an online community. Many of these fundamental characteristics remain in MetaMAP despite evolution of the design through multiple stages with diverse collaborators.

#### (1c) Testing

**Fig. 6:**
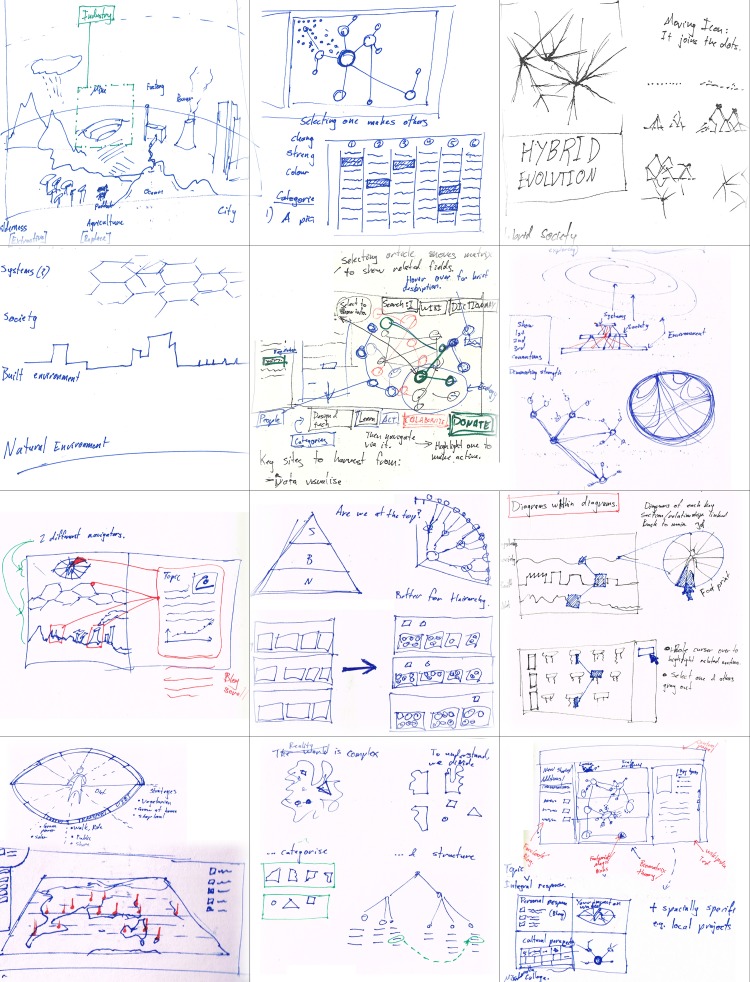
Collection of concept sketches developing ideas for MetaMAP. Recurring themes included social–ecological systems, exploration of different hierarchies among parts, landscape metaphors, graphic means of managing complexity and tools to help users navigate an ecosystem of knowledge

Throughout this early stage, it was important to identify and test a wide range of possible approaches before committing to one. This involved creating many hundreds of drawings exploring different ideas (Fig. 6). Sketching helped us to develop and critique ideas rapidly through many iterations. Representing ideas visually allowed us to see potential consequences which we may have otherwise overlooked. This testing often involved considering the challenge from one perspective, drawing ideas which came to mind, and then critiquing them from several other perspectives. This is commonly known as a ‘conversation with the self via the pen’ (e.g. Kennedy-Clark 2013). The growing collection of drawings provided a point of comparison for later developments. These methods continued to be used throughout the entire design process for developing and testing ideas.

#### (1d) Reflections

This stage also provided insight on issues which would shape future developments. There were considerable challenges in representing many abstract sustainability concepts vividly. As such, the designs constantly shifted between highly abstract representations of sustainability ideas and more tangible ‘landscapes’ which expressed sustainability issues through metaphor. The metaphorical landscapes were based on familiar elements of built and natural environments and fostered rapid understanding.

We found that systems approaches were much better suited to synthesising and understanding sustainability issues than hierarchical frameworks. Systems models are valuable for understanding relationships, but without an underlying structure, they can be very difficult to navigate. As such, sustainability scientists and practitioners may benefit greatly from organising systems models, but how? An organising framework should help users to understand them rapidly, build familiarity over time and inspire insight into higher order phenomenon. On the other hand, any such underlying framework would risk excluding ideas which are important for sustainability. Different disciplines and cultures have different ways of understanding the relationship between people and nature. We realised that to bring these together into a common framework would require input from people of different backgrounds.

### Stage (2) Student workshop

#### (2a) Problem/opportunity framing

This stage focused on developing the conceptual framework used to organise and navigate the envisaged ‘digital world’ of sustainability ideas and action. Following Stage 1 and an extensive review of literature and precedents, we developed a manifesto which set out the long-term ambitions of the project (See Text Box 1). This informed a design brief for the project (not shown) which set objectives for several aspects of MetaMAP including: knowledge transfer and development, conceptual frameworks and concepts, users and how they interact, social empowerment, content, communication, interface, governance and management, aesthetic and technical requirements and fostering social-environmental impact beyond the platform. It was continuously refined following insight gained in later stages.

**Figure:**
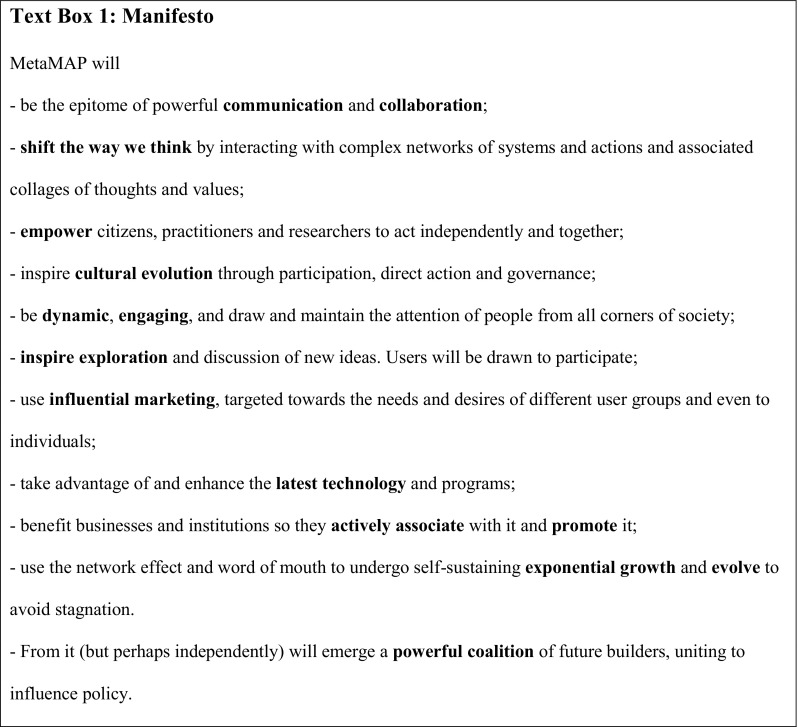


#### (2b) Solution development

**Fig. 7:**
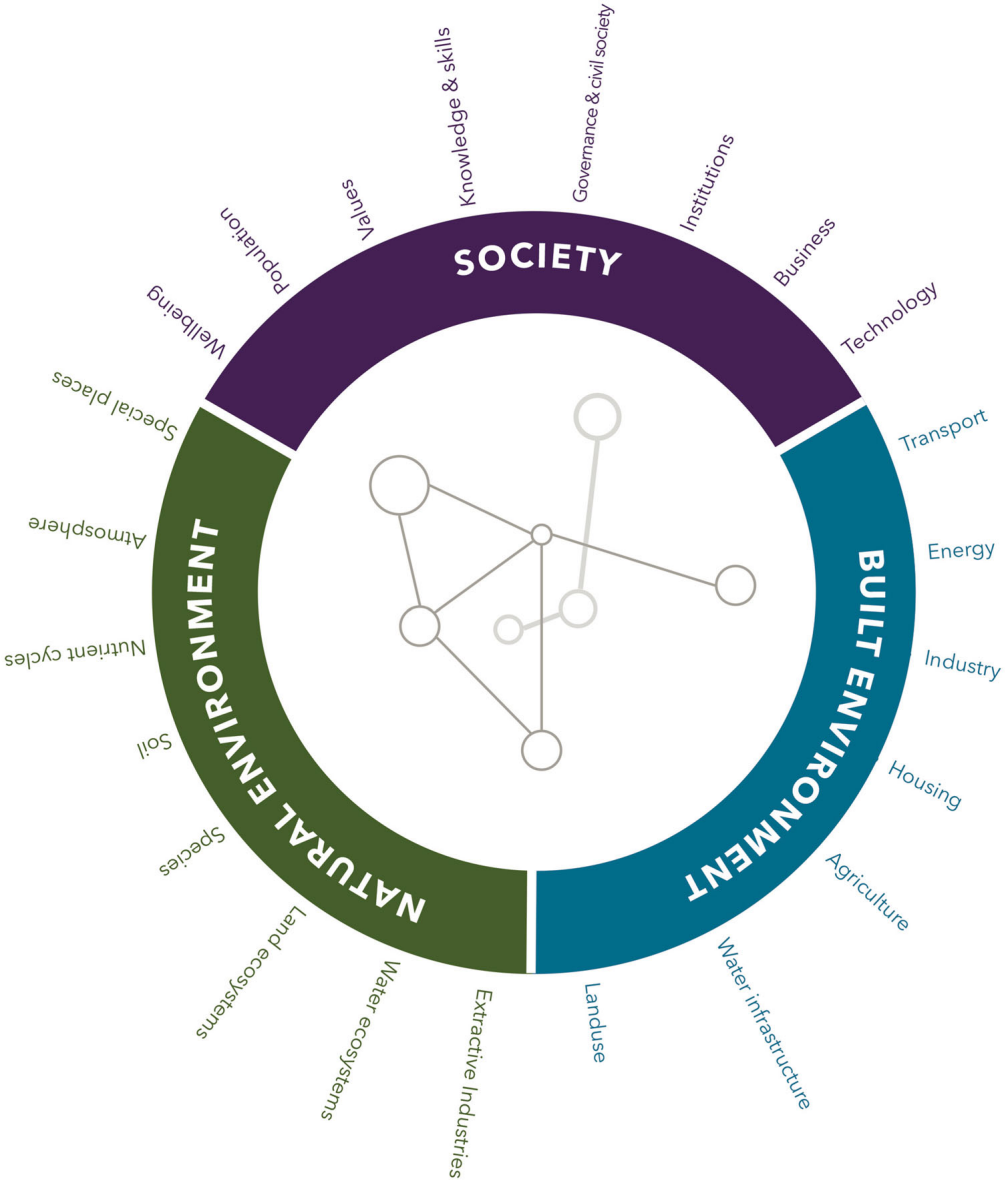
MetaMAP circular prototype

Here, we focused on refining the framework used to give structure to systems models. It needed to be intuitive, and encompass a wide diversity of perspectives on sustainability issues. We developed a circular framework which was overlaid by concept maps of sustainability challenges. The circle was divided into three primary realms: the natural environment the built environment and society. These were further subdivided into categories as presented in Fig. 7. These realms and categories helped to locate particular issues within a concept map.

#### (2c) Testing

We ran a 2 h workshop to test the effectiveness of the framework for learning and guiding exploration of sustainability ideas. We investigated if participants could understand the framework. Did it help them to think in systems? Did it aid collaboration? Did it facilitate insight? The workshop involved 104 s year Architecture students with no prior education in systems theory, and no prior tertiary education in sustainability. Participants selected a familiar building and considered the impacts that it had on its broader social–ecological context. They represented each impact with a labelled arrow connecting the building in the centre to the category it affected in the edge. Facilitators noted the students’ insight of how the building influenced the social–ecological system and compared it with student insight from previous activities without the framework.

#### (2d) Reflections

We found that the structured categories and realms were especially valuable in helping participants tap to into their existing general knowledge and consider issues they may have otherwise overlooked. By working together on a single framework, participants could build on each other’s ideas and contribute to a greater shared understanding of the topic. The structured framework and semi-guided process also helped participants to gain insight on higher order properties of the system. For example, one group drew a circle around the entire system and said “When you think deeply, everything is impacting on each other!”—a potentially transformational paradigm shift in young architects. It also helped them to apply systems thinking rapidly without prior knowledge.

The arrows showing relations were useful to guide thinking during the task, but were difficult to translate later if not well noted. A more intuitive system would allow faster understanding by a broader audience, easier application, and more developed high order thinking in less time. We realised that improving the intuitiveness of the framework should be a priority throughout the project.

### Stage (3) Ecovillage case study

#### (3a) Problem/opportunity framing

Next, we broadened the literature review and conducted a number of expert interviews which highlighted the importance of scale when addressing sustainability challenges (Wu 2013). It is often necessary to examine relationships among issues occurring at different physical scales, i.e. ‘think globally, act locally’. In addition, purely objective approaches to sustainability separate people from the systems they influence. In contrast, other approaches which synthesize objective (user outside the system) and subjective (user within the system) perspectives can be more comprehensive and empowering (e.g. Integral theory) (Brown 2007). This would be a valuable addition to a framework for organising diverse content on sustainability. We also continued to increase the intuitiveness of the framework by representing abstract ideas through familiar visual metaphors.

#### (3b) Solution development

**Fig. 8:**
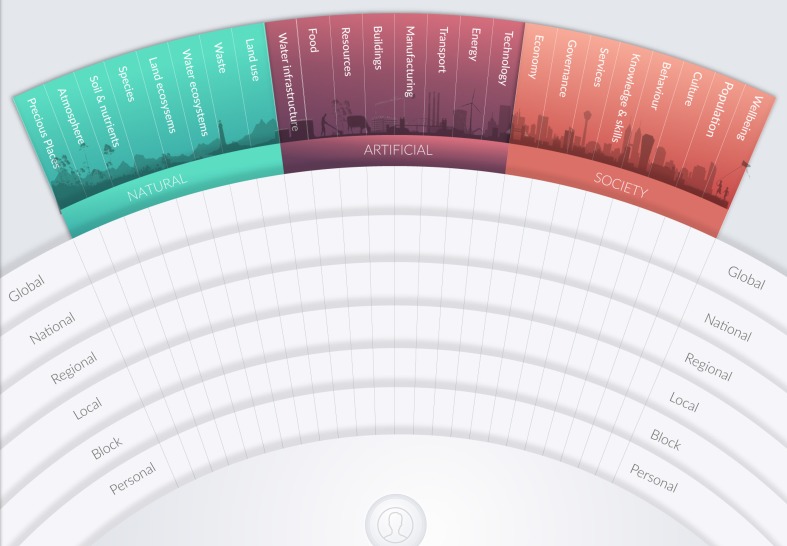
MetaMAP prototype

We advanced the design substantially in response to our growing understanding of the challenge and potential solutions as in Fig. 8. A physical scale was introduced ranging from personal up to global. To aid understanding, the framework was represented as a portion of a globe with smaller scales in the foreground and larger scales on the distant horizon. The horizon edge was visualised as a stylised landscape ranging from natural through industrial to urban. This reinforced both the physical scale and different elements of the social–ecological system. The individual user was represented in the foreground from which point connections could be made to the system they are examining. The MetaMAP framework is intended to support the SDGs, but at this point we chose not to include the 17 goals explicitly to reduce complexity for unfamiliar users.

#### (3c) Testing

**Fig. 9:**
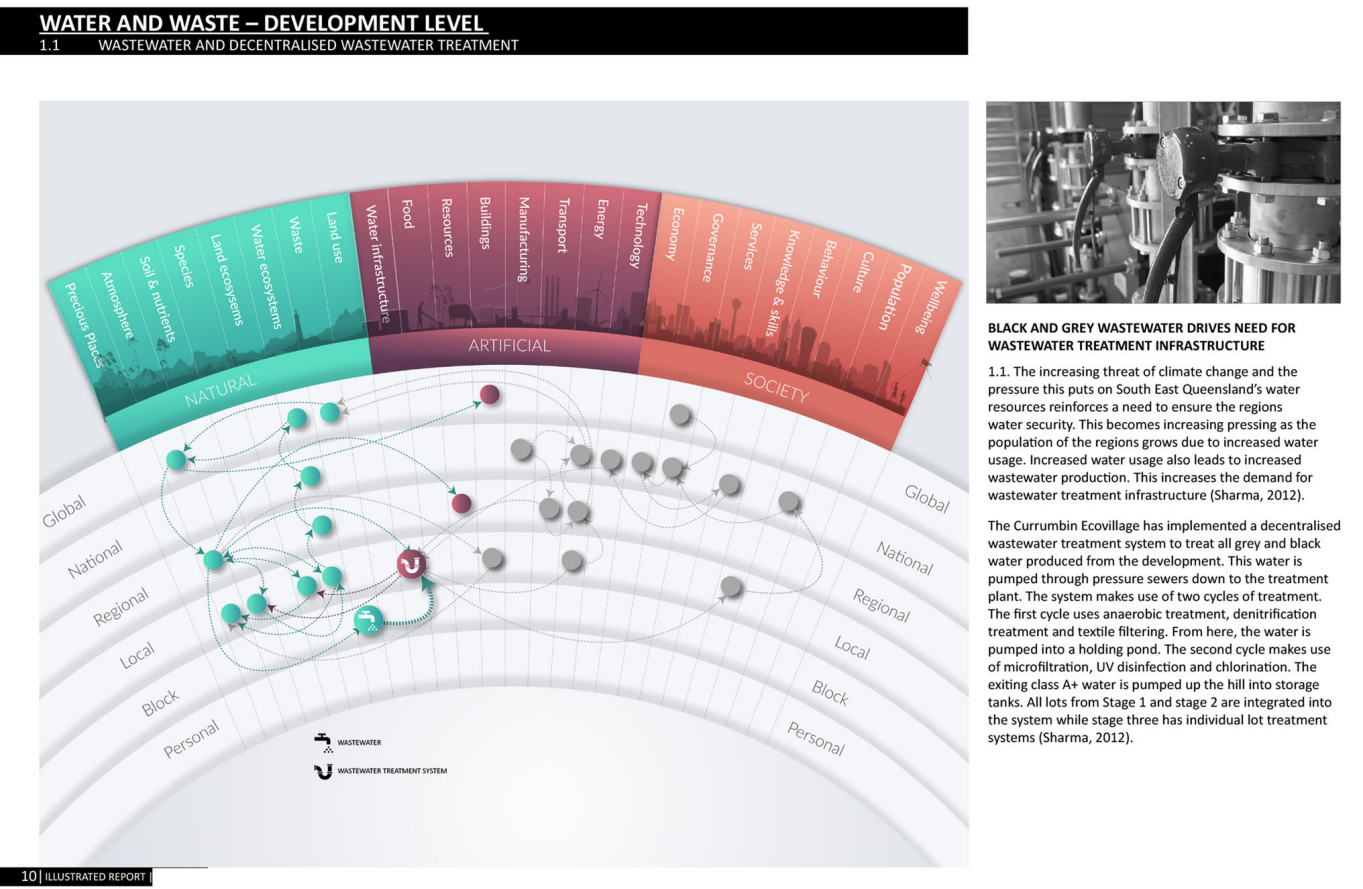
Page from student assignment applying MetaMAP prototype to understand how water and waste systems within the Ecovillage shape the broader social–ecological system across scales. Each page of the assignment highlighted a subset of issues (crimson on the left) and described how they relate (text on right). Source: Rehn 2016 (with permission)

The next cycle of testing sought to understand how the framework might help to guide learning and exploration of a complex sustainability-driven initiative. Could it help people unfamiliar with systems approaches to visualise interactions across scales and sectors? Over 6 weeks, 15 Masters of Architecture students used the MetaMAP framework to conduct a holistic case study of Currumbin Ecovillage: a community in the Gold Coast hinterland seeking to live sustainably and regenerate the local ecosystems (O’Callaghan et al. 2012). These students had extensive design training but no prior education in systems approaches and little sustainability education. Each student selected a different aspect of the Ecovillage to examine over the 6 weeks period. For example, some studied water or energy systems while others considered construction materials or community culture. Students visited the site, conducted independent research and collaborative sessions and an oral presentation. Throughout the project, the MetaMAP framework was used to describe relationships between the Ecovillage and different aspects of our social–ecological system across scales (e.g. Fig. 9). In a final workshop, the class reflected on the project and critiqued the framework.

#### (3d) Reflections

As a whole, students took several sessions to understand the MetaMAP framework and systems thinking approach. Once familiar, however, all 15 students applied the framework with great enthusiasm and intellectual rigour. All students identified various paths through which the Ecovillage shaped the natural environment, the built environment and society. This helped them to examine details of the project in the context of the broader social–ecological system. All identified cross-scale interactions and came to appreciate how their role as designers contributed to global challenges. One said that without the framework “I wouldn’t have known where to start”. Several students identified feedback loops in their system without being introduced to the concept. Students used the MetaMAP framework in their presentations which proved valuable in communicating clear narratives through complex systems—they mapped the path of their argument visually. Many students developed unique visual methods for describing phenomena they identified in their system (e.g. ‘ripple effects across scale, sub systems, overall impacts, effects of time). Despite this, the sheer complexity of the systems led to concept maps which were difficult for others to follow. This visual complexity needs to be managed carefully. These tests revealed opportunities to develop guided processes to help unfamiliar users create concept maps. They also reinforced that understanding the specific context of use is essential for designing sustainability initiatives.

### Stage (4) Framework comparative analysis

#### (4a) Problem/opportunity framing

MetaMAP had so far proved highly effective in sustainability education. However, by focusing on parts of the social–ecological system and how they relate, some important emergent properties were left out of the framework. These included concepts such as carrying capacity, resilience and ecosystem services. Analysis of the existing conceptual frameworks of sustainability identified other important perspectives on sustainability which we then aspired to incorporate into MetaMAP.

#### (4b) Solution development

**Fig. 10:**
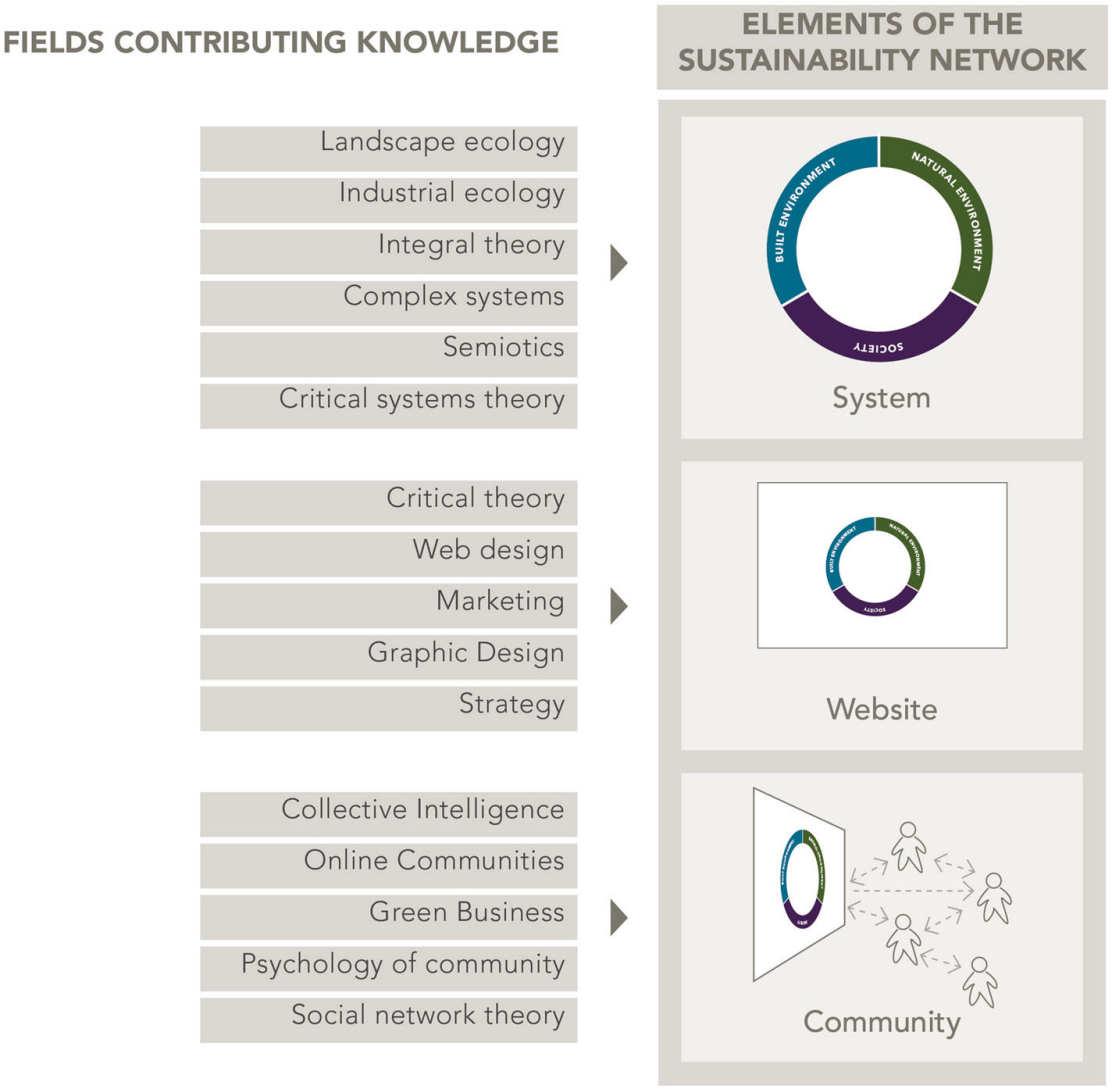
Nested elements of MetaMAP and the disciplines which informed their design

During this time, our understanding of MetaMAP expanded into the digital environment. We now envisaged three nested elements: (1) the underlying conceptual framework and concept maps developed through previous stages; (2) a digital interface through which users navigated content visually; and (3) a diverse community of sustainability ambassadors collaborating through online networks. Figure 10 describes these nested elements and several academic and practical disciplines which contribute theory to the design.

#### (4c) Testing

**Fig. 11:**
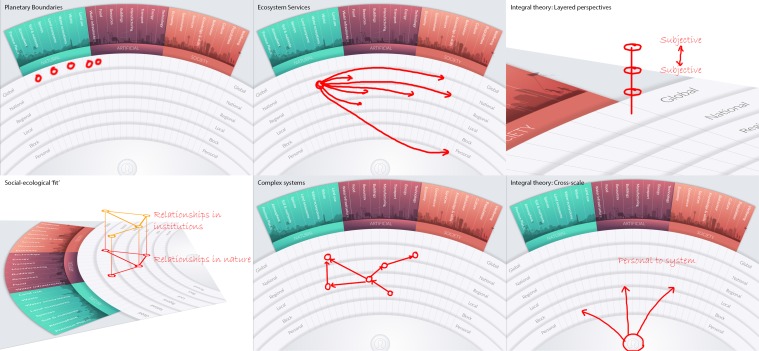
Examples of MetaMAP expressing existing conceptual frameworks of sustainability

Seeking greater comprehensiveness, we next compared MetaMAP with existing conceptual frameworks related to sustainability to identify important concepts which it so far neglected. This exercise was carried out visually as diagrams can be an effective way of expressing paradigms of thought and the concepts of which they are composed. First, we compiled a collection of leading conceptual frameworks. We sourced these from prominent institutions (e.g. Millennium Ecosystem Assessment 2005; Rockström et al. 2009) and Sustainable Lens (Mann 2011). We then attempted to map each framework onto MetaMAP as shown in the examples in Fig. 11.

#### (4d) Reflections

The majority of frameworks were easily transcribed onto the MetaMAP framework. These included Planetary Boundaries, the United Nations Sustainable Development Goals (SDGs), Ecosystem Services, People-Profit-Planet (Sosik and Jung 2011) and Complex Systems (van Kerkhoff 2014) among many others. Other Frameworks were able to be mapped only by developing the design of MetaMAP. For example, Social–Ecological Fit examines how relationships between people correlate with relationships among the elements of the social–ecological system which they manage (Guerrero et al. 2015). We were able to support the concept of Social–Ecological Fit in MetaMAP by adding a new ‘layer’ for people overlaying the social–ecological system (Fig. 11, bottom left). The concept of time remained important yet challenging to include in MetaMAP. Eventually we solved this by ‘tagging’ each relationship link in a concept map with the duration it takes to unfold.

### Stage (5) Researcher workshops

We then sought to test MetaMAP with a more experienced user group and greater diversity of sustainability projects.

#### (5a) Problem/opportunity framing

MetaMAP had proved to be quite comprehensive in the previous stage of testing, and able to synthesise most of the frameworks and concepts tested. Most of the issues it failed to include were social processes which are important for sustainability rather than elements of the social–ecological system. For this next stage, the underlying conceptual framework remained unchanged, but we developed guided processes to help new users apply it effectively.

#### (5b) Solution development

We expanded the previous model of MetaMAP which included three nested elements to include a fourth element: guided process of mapping sustainability issues. We conceived of this process as a combination of workshop facilitation and strategic design methods. This allowed us to incorporate a suite of important sustainability theory related to social processes for achieving sustainability goals which, until now, had been overlooked.

#### (5c) Testing

We then sought to test MetaMAP with a more experienced user group and greater diversity of sustainability projects. As before, we aimed to identify unforeseen problems but also had guiding questions: could we increase the speed at which new users could understand and apply MetaMAP? Could it cope with a wide variety of applications? Could it help users to position their work in the context of broader sustainability initiatives such as the United Nations Sustainable Development Goals?

**Fig. 12:**
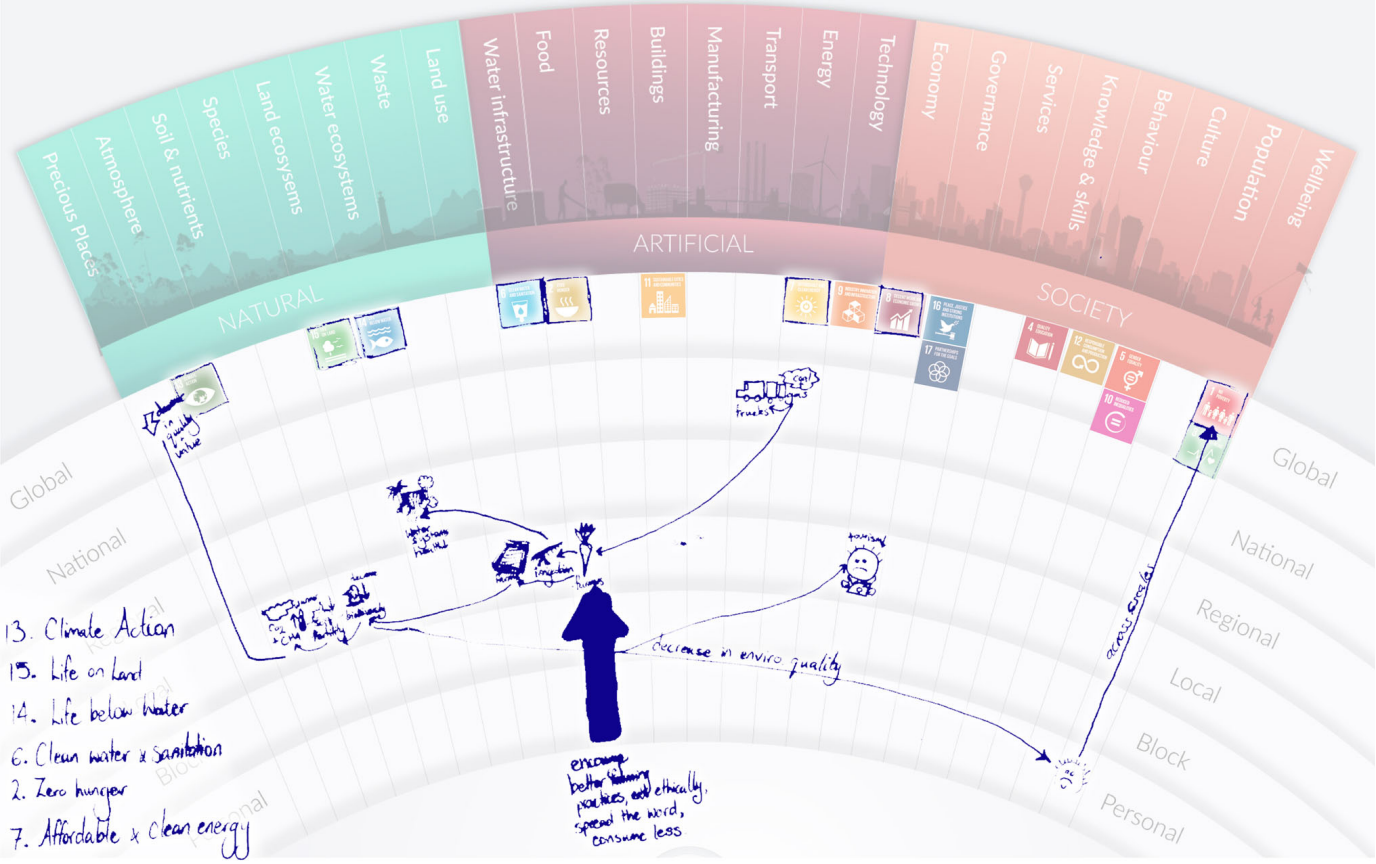
Workshop application of MetaMAP showing point of leverage over food system and implications for the SDGs

To examine these issues, we ran a series of three, 2 h workshops with a total of 26 participants. To ensure that MetaMAP could support a wide diversity of perspectives on sustainability we engaged participants from many different disciplines with experience ranging from undergraduate to experienced researchers. Workshops began with a brief introduction to the MetaMAP framework. The session leader projected the framework onto a whiteboard and drew an example concept map with input from participants. Each participant then mapped their current sustainability project on a sheet of transparent paper overlaying an A3 print of MetaMAP. The projects were diverse including: impacts of farming on creeks in Otago (Fig. 12), improving bicycle networks in Dunedin, social housing policy for children’s wellbeing and water scarcity as a factor in intrastate conflict. The mapping loosely followed a guided process shown in Text Box 2. Participants then discussed their work in groups of two or three. They overlaid their transparent sheets to compare models and identify issues of common interest. For example, farming practices in Otago and community forest regeneration schemes can both support the health of local creeks. Finally, participants placed their conceptual model over a version of MetaMAP which had the 17 United Nations Sustainable Development Goals (SDGs) mapped on it. This helped them to identify which SDGs their project might contribute to and how.

**Figure:**
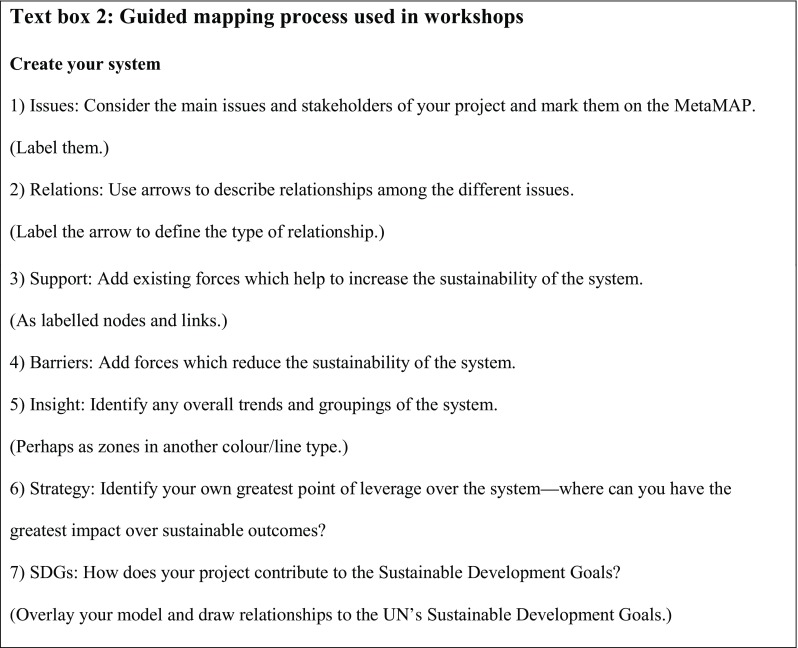


#### (5d) Reflections

The diversity of the three workshops provided a rich foundation for reflective learning. By introducing principles of design thinking, the guided process helped turn a previously descriptive or reactive activity into a proactive one. Instead of merely describing a system disconnected from the participants or identifying problems, MetaMAP helped users to see themselves in the system. Many participants identified strategies within their power which could help to transform the system towards greater sustainability. When a framework was not used, participants had difficulty organising concepts in a meaningful way. This limited higher-level insight.

Many participants found MetaMAP useful for self-reflection as it helped them to “…analyse our own views points and show relationships between them.” Some expressed that the exercise helped them to frame their research in a broader context. Another “…found it valuable to conceptualise how different issues interact and the relationships between them, however, it is difficult to map the full complexity of this.” The visual space consumed to represent an idea limited the number of ideas which could be seen (and hence considered) together. A digital environment would allow for a greater density of information with easily collapsible and expandable content. This would facilitate more complex and subtle thinking necessary for addressing sustainability issues. The large projector screen used in the introduction proved especially valuable for collaboration which highlights opportunities for educational, commercial and institutional applications.

By connecting their own projects with the SDGs, some participants (especially younger ones) were greatly empowered—one was visibly moved. However, many had a limited understanding of their own opportunities in driving systemic change. The geographic scale within the MetaMAP framework proved insufficient for many disciplines. For example, some from social sciences and humanities backgrounds requested scales of human interaction such as individual, family, community, society, and civilisation. Some participants found that within the system they studied, stakeholders were disconnected from the issues they manage—a critical challenge for sustainability practitioners (Guerrero et al. 2015). This led us to develop methods for improving ‘Social–Ecological fit’ in later iterations. Opportunities remain for developing more guided processes based on collaborative design and facilitation methods.

### Outcomes of the design process

We now provide a brief overview of MetaMAP in its current state and its value for achieving SDGs (Fig. 1). A detailed description with worked examples and further applications can be found in (Maher et al. 2018a). A blank version of the MetaMAP framework is provided as an appendix for use by readers. MetaMAP is a graphical tool which supports collaborative investigation and design for achieving SDGs. It helps diverse users to integrate their thinking, understand sustainability challenges holistically, and develop well-integrated solutions. MetaMAP is based on a new high-level conceptual framework which gives structure to social–ecological systems built collaboratively by interdisciplinary teams. The framework synthesises important concepts drawn from multiple schools of thought including: Social–Ecological systems (e.g. Partelow 2015), Planetary Boundaries (e.g. Rockström et al. 2009), Design thinking (e.g. Glanville 2007), Integral theory (e.g. Brown 2007) and Ecosystem Services (e.g. Abson et al. 2014) among others. This underlying framework is represented visually as a ‘landscape of ideas’. Over this ‘landscape’, users add concepts—icons which represent important components of the social–ecological system being investigated. The ‘landscape’ locates concepts based on their scale (the *y*-axis ranging from personal to universal) and how they may be categorised in the social–ecological system (*x*-axis grouped into three realms: the natural Environment, the built environment and society). Users link concepts using lines and arrows to represent how they relate (e.g. increases, decreases, restricts, etc.). As interdisciplinary teams contribute concepts and links, they build up a conceptual model of the social–ecological system being investigated. The framework helps users to consider a wide variety of issues and gain insight into patterns and trends in the system. These big picture issues can be represented as notations and groups. Common high-order concepts (e.g. resilience and synergy) are located in the ‘emergent properties’ and ‘guiding principles’ boxes above the landscape. The SDGs (or other guiding frameworks) can be located on the landscape to help users see how the system they influence may support or compromise the SDGs.

We have designed a digital platform which uses the MetaMAP framework to organise and navigate diverse content contributed by a community of users from around the globe. We are currently seeking collaboration to support its development.

### Applications and benefits for different users

MetaMAP helps users to achieve sustainability goals in a number of complementary ways. The structure and application of the MetaMAP framework help users gain insight by seeing relationships among parts of the natural environment, built environment and society across multiple spatial and temporal scales. It provides an inclusive framework to help people from different backgrounds integrate their diverse perspectives on sustainability issues into a common understanding. Decision makers can use MetaMAP to help understand complex challenges, identify strategies with synergy and design well-integrated solutions. Researchers can use MetaMAP to identify gaps in knowledge and communicate the broader implications of their research. Learners can use MetaMAP to explore diverse content on sustainability and understand connections between seemingly isolated issues.

## Design principles for achieving SDGs

Examining the process of designing MetaMAP sheds light on important design principles which help to solve wicked problems and achieve SDGs. Design brings with it a particular way of understanding; “…a way of looking at the world and reshaping it, a way of generating knowledge through creation” (Overbeeke and Wensveen 2003). To support this, design approaches can help people to understand complex situations in new ways which generate new types of solutions. This is of critical value for transforming our social–ecological system to be more sustainable (Westley et al. 2011). When used alone, traditional methods which focus on optimising existing circumstances will remain unable to generate the degree of change needed to achieve sustainability goals. We now discuss some design principles used in this project and their broader implications for achieving SDGs (Table 2).

**Table 2:** Design principles, examples from the design of MetaMAP, and how MetaMAP helps users to apply the principle in practice

Design principle	Example methods from the design of MetaMAP	How MetaMAP helps users to apply it
Broad problem framing supporting multiple goals	Reframing the brief Design manifesto Guiding questions Unconstrained user feedback Diverse interdisciplinary collaboration	Scales and categories of framework expands conception of challenge Links to several SDGs Guided processes
Maximise synergy, minimise compromise	Reorganising ideas using concept maps, pinboards and sketches to identify strategies with synergy Focus on opportunities	Guided processes Points of convergence in concept maps highlight synergy Framework incorporates emergent properties
Integrating diverse perspectives	Interdisciplinary workshops, interviews, literature review Imaginative roleplaying during design Diverse case study applications	Framework synthesises divergent perspectives Visualising cross-scale interactions Users build concept maps to integrate ideas Guided process of collaboration
Thinking visually	Sketching Examining precedents Workshop exercises	Graphical interface stimulates mental visualisation
Multiple feedback loops	Sketching ideas Multiple cycles of critical reflection Stakeholder testing Agile attitude of designers	Visuals aid critical reflection; Guided processes with repeated reflection Interdisciplinary teams with different perspectives

### Broad problem framing supporting multiple goals

Whereas traditional approaches to research narrow their focus towards strictly defined objectives, design approaches often lead to a broader understanding of problems and potential solutions (Faste and Faste 2012). This helps design researchers to challenge assumptions and to remain open to shifting their objectives in the light of new understanding. Achieving SDGs routinely involves conflicts among stakeholders with different values and goals. Framing sustainability projects narrowly puts different goals into opposition and increases these conflicts. Alternatively, design approaches which broaden their problem framing can help to find a ‘higher common purpose’ among stakeholders and reduce conflict (Patel 2005). More inclusive conceptual frameworks can also help sustainability researchers and practitioners to take a broader perspective.

### Maximise synergy, minimise compromise

In order to create well-integrated results, designers seek to identify strategies with synergy—where a single approach can help to achieve multiple contradictory goals simultaneously (Glanville 2007). In architecture for example, a line of columns in a building might simultaneously (1) support a roof, (2) define a path, (3) provide privacy, (4) form a space, (5) embellish a façade, and (6) express wealth and power. Where this is successful, the whole becomes much more than the sum of its parts. Any attempt to quantify its value or judge it by a single predetermined metric undervalues it and distorts reality. This highlights the risk of relying on many common tools and approaches to achieving SDGs. Building on synergy can also help to minimise conflict. For example, if two goals, stakeholders, or SDGs are typically in conflict, a design approach would be to seek atypical situations with the potential to minimise/avoid/reverse the conflict. However, developing synergetic strategies is challenging, requiring designers to constantly shift their own perspective. Each new way of looking at a particular design proposal provides new insight on its shortcomings and opportunities for its development.

### Integrating diverse perspectives

Achieving sustainability goals entails transforming our social–ecological system so that many parts work in harmony. This is challenging, however, as the perspective of any one individual or discipline focuses on some issues while neglecting others. As such, no one perspective or discipline working in isolation can be expected to develop well-integrated sustainability initiatives. Failing to consider an important perspective may lead to blind spots in a proposal which become likely points of failure. To avoid this narrow mindedness, a core question of design thinking is ‘what am I missing?’.

When making decisions, many people employ a number of techniques to avoid the problems of single perspectives, such as listing pros and cons or Edward de Bono’s ‘six thinking hats’ (De Bono 2017). Designers have other methods. The RtD case study above introduced the perspectives of different disciplines through collaborative activities such as interviews and workshops. Visual methods can also help people take on multiple perspectives by representing the same idea in different ways (Agrawala et al. 2011).

In designing MetaMAP, this deliberate shifting of perspective took many forms. Sometimes it involved focusing on one objective, then another. Roleplaying helped us to imagine how different potential users might respond to some aspect of the design. Sometimes we extended this by imagining entire scenarios involving particular users in a particular context using MetaMAP to pursue their own unique interests. This helped us to tailor the current prototype to specific context and applications. Knowing when to apply each approach for maximum benefit requires constant self-reflection and is among the most difficult design skills to learn.

This approach has many benefits for achieving SDGs as each new way of looking at a project provides new insight into challenges and opportunities. A problem which seems impossible from one perspective may be solved easily from another. Sustainability initiatives developed by integrating diverse perspectives can also expect to benefit from wider support among stakeholders.

### Thinking visually

Many sustainability challenges are too complex to hold it in one’s mind, so they must be visualised to be considered holistically. Diagrams can help sustainability researchers to identify existing and possible relationships between parts and to “…understand the research in totality and…freely manipulate and associate individual pieces of data” (Kolko 2009). Similarly, holistic frameworks can help decision makers to see the details of a project in a wider context. That way, decisions at a small scale can contribute positively to the project at large. Achieving SDGs involves complex relationships within social–ecological systems. Visual methods can represent these non-linear circumstances far more clearly than words. Communication is a critical limit in developing interdisciplinary sustainability initiatives (Godemann and Michelsen 2011) and visual methods can help to bridge disciplinary silos by providing a common language.

Many disciplines contributing to sustainability use specialist visual tools such as geographic maps and data visualisation. Like design tools, these visuals help researchers to gain insight into complex issues. However, to aid design thinking, visual tools should allow users to represent diverse ideas and manipulate them freely. Despite their value in sustainability research, many academic publishers restrict the use of visuals to the minimum required to support the text. Instead, publishers should seek the optimal number and type to communicate complex issues vividly to a wide audience.

### Multiple feedback loops

In sustainability as in other wicked problems, it is rarely possible to see a direct path to a successful outcome. The many unknowns and unforeseen opportunities mean that any approach to creating a solution must be adjusted along the way. The more cycles of action and reflection, the faster an effective strategy can be identified. To achieve this, design approaches employ a fractal of feedback loops—often many thousands in a single project. At the largest scale, lessons learned from each project inform the next. Within a project, reflecting on progress in each stage shapes the activities of the next. When testing ideas collaboratively, each person’s response may guide future design. Each new perspective a designer takes on provokes the next idea. Seeing each new drawing informs the next, and even within a single sketch, drawing and seeing each line helps guide the next. Each feedback loop is a new opportunity to steer the project towards outcomes which will be effective in the complexities of the real world. Applying a fractal of feedback loops to the pursuit of sustainability goals delivers initiatives with greater synergy, fewer objections, providing more benefits and better adapted to their unique contexts.

The scientific method requires that each step must be justified objectively and to the satisfaction of external reviewers. However in design, and when seeking transformational change generally, it is essential to suspend judgement (de Bono 1970) so that new strategies can be tested despite initial uncertainty and limitations. Reliability in design is generated not through concrete incremental advances, but by insightful leaps refined through numerous cycles of critique, editing and development (Dorst 2011).

## Future work

The SDG tools compendium (Asian Development Bank 2018) introduces 134 tools for helping to address environmental SDGs in Asia. These are sorted into 17 different categories and support critical endeavours including analysis, budgeting, stakeholder engagement, building scenarios and developing measurements. However, none of the 134 tools are explicitly for designing sustainability initiatives using established design principles and methods. Only one provides an excellent yet brief and general introduction to design thinking (Elmansy 2017), and it does not provide tools tailored for designing sustainability initiatives.

MetaMAP can help users to apply design approaches to achieve SDGs in a wide variety of contexts. We presented MetaMAP in Stockholm at Resilience 2017 (Maher 2017b) and the International Conference on Sustainability Science (ICSS) (Maher 2017a). Afterwards, we were approached by people from academia, Non-Government Organisations and governance backgrounds seeking to apply MetaMAP in diverse contexts including:

- Resilience planning in South and Southeast Asia
- Multiple ecosystem services and urbanisation around Shanghai
- Air pollution in China
- Facilitating Academic‐Industry collaboration in sustainable agriculture
- Community development planning in Guyana
- Cross‐scale issues in health systems
- Organisational strategy for sustainability
- Communicating how SDGs are being addressed in India
- Sustainability education

Requests for these broad application domains demonstrate both the flexibility of the MetaMAP system and strong demand for the benefits it provides. Applying MetaMAP in contexts such as the above is required to enhance it and develop a digital platform. This response also demonstrates the potential value of the RtD process that created it.

## Conclusion

In this paper, we discussed the value and limitations of new tools for achieving SDGs. This identified a pressing need for: (1) a stronger integration of design approaches with sustainability science; and (2) tools which help researchers and practitioners apply design thinking to develop sustainability solutions. We provided an overview of Research through Design methodology then examined a case study of its application: the process of designing MetaMAP. This involved five stages, each including: (re)framing the problem/opportunities, designing possible solutions, testing them collaboratively and reflecting critically. We concluded the case study by providing an overview of MetaMAP—a graphical tool for collaborating to understand social–ecological systems holistically and design well-integrated sustainability initiatives. Reflecting on this case study, we presented some fundamental design principles which were demonstrated through the RtD case study and their value for achieving SDGs.

Integrating design and sustainability science hold much value for transforming our social–ecological system to achieve SDGs. However, there are some significant challenges in doing so. Many aspects of a Research through Design project cannot be predetermined (Moloney 2015) which provides challenges for traditional research grants. There is a critical shortage of literature and guidance on design approaches for sustainability research. Design approaches to achieving SDGs can be advanced by (1) collaborating and learning from those experienced in creative design methods; (2) expanding opportunities for publishing creative explorations and visioning (Wiek and Iwaniec 2014); and (3) applying design methods to SDGs and sharing the results and process.

More specifically, MetaMAP can help researchers, practitioners and educators to understand the context of sustainability challenges more holistically and design initiatives. MetaMAP necessarily contains some compromises including: the categories, scales and guided process may be unsuited to some applications; and the apparent complexity of the framework may require practice and/or training to apply. Refining the usability and effectiveness of MetaMAP requires further application by people seeking to achieve SDGs in diverse settings. We are pursuing to develop MetaMAP into a digital platform for collaborating across disciplines and borders to achieve SDGs. This requires substantial testing, expertise and resources for which we are currently seeking collaboration. Combining the methods of science and design can help to develop more innovative, better integrated and truly transformational initiatives for sustainability. We encourage readers to apply design approaches and the MetaMAP graphic tools to their own unique sustainability projects and share their findings.

## Electronic supplementary material

Below is the link to the electronic supplementary material.

Supplementary material 1 (jpeg 364 kb)
