# Supplementary figures and images for: Integrating design thinking with sustainability science: a Research through Design approach
Source: Sustain Sci. 2018 Aug 20;13(6):1565–87. doi: 10.1007/s11625-018-0618-6 (PMC6267153; doi:10.1007/s11625-018-0618-6)

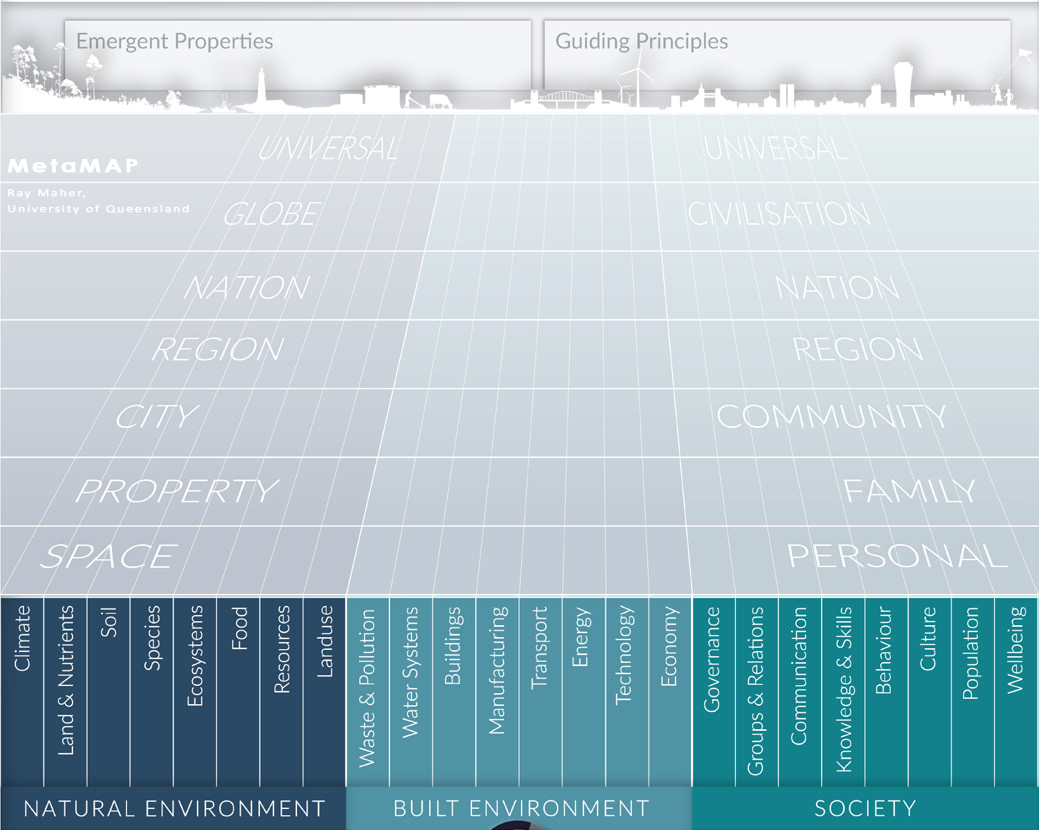

Supplement: Supplementary file 1 — Supplementary material 1 (jpeg 364 kb) [file 11625_2018_618_MOESM1_ESM.jpg]
